# Adverse events associated with third-generation cephalosporins: Analysis of the FDA adverse event reporting system database

**DOI:** 10.1097/MD.0000000000045778

**Published:** 2025-11-07

**Authors:** Nehad J. Ahmed

**Affiliations:** a Department of Clinical Pharmacy, College of Pharmacy, Prince Sattam Bin Abdulaziz University, Alkharj, Saudi Arabia.

**Keywords:** adverse events, cephalosporins, FAERS, reporting

## Abstract

Third-generation cephalosporins are widely used and can cause side effects like nausea, vomiting, loss of appetite, and stomach pain. Hypersensitivity reactions and drug-induced immune hemolytic anemia may also occur. This study investigates adverse events associated with these drugs using the FDA adverse event reporting system database. This descriptive study assessed adverse events linked to third-generation cephalosporins reported to the Food and Drug Administration. The data was analyzed descriptively and presented as numbers and percentages in tables, each corresponding to a specific third-generation cephalosporin. The most reported adverse events of cefotaxime were pain, eosinophilia, and ineffective drugs. Ineffective medication, pyrexia, and sepsis were the most frequently reported adverse effects of ceftazidime. The most reported events of cefdinir were diarrhea, drug hypersensitivity, and rash. The most reported events of ceftriaxone included off-label use, ineffective drugs, and eosinophilia. The most reported events of cefpodoxime included pyrexia, pruritus, and diarrhea. The most reported events of cefoperazone were a decreased prothrombin level, hemorrhage, and pyrexia. The most reported adverse events of cefixime included diarrhea, clostridium colitis, and ineffective drugs. This study highlights that gastrointestinal issues, hypersensitivity reactions, and inadequate medication responses are the most commonly reported adverse effects of third-generation cephalosporins. Despite their effectiveness in treating various infections, healthcare professionals must remain vigilant about the potential side effects of these drugs.

## 1. Introduction

Cephalosporins are β-lactam antibiotics classified into 5 generations based on their antimicrobial spectrum and discovery timeline. All generations treat infections like pneumonia, meningitis, and skin/soft tissue infections. First-generation agents primarily target gram-positive cocci and some gram-negatives (Escherichia coli, Proteus, Klebsiella).^[1]^ Second-generation cephalosporins cover Haemophilus influenzae, Moraxella catarrhalis, and Bacteroides. Third-generation cephalosporins shift toward Enterobacteriaceae, Neisseria, and H. influenzae with reduced gram-positive activity. Fourth-generation agents add β-lactamase-resistant gram-negative coverage, while fifth-generation cephalosporins target Methicillin-resistant Staphylococcus aureus and penicillin-resistant pneumococci.^[1]^

Third-generation cephalosporins are the most clinically used, featuring modified C7 acylamido chains (e.g., ceftriaxone, ceftazidime). While broad-spectrum, they’re more effective against resistant gram-negatives than first-gen drugs but weaker against Streptococci/Staphylococci.^[2]^ Third- generation cephalosporins show superior β-lactamase resistance against Klebsiella, H. influenzae, and E. coli. Only cefoperazone and ceftazidime cover Pseudomonas. Notably, they remain effective for empirical spontaneous bacterial peritonitis treatment despite increasing gram-positive prevalence.^[2]^

Cephalosporins are generally safe with low toxicity. The most frequent adverse effects are gastrointestinal (GI) (nausea, vomiting, anorexia). Hypersensitivity reactions (rash, urticaria, angioedema) occur less frequently, predominantly with first- and second-generation agents. Rare but serious complications include drug-induced immune hemolytic anemia, particularly associated with ceftriaxone and cefotetan.^[1]^ Cephalosporins with a methyl-tetrazole-thiol side chain (e.g., cefamandole, cefoperazone, moxalactam) can cause disulfiram-like reactions by inhibiting aldehyde dehydrogenase. Additionally, some cephalosporins impair vitamin K metabolism, increasing bleeding risk (hypoprothrombinemia). Third-generation agents are also associated with pseudomembranous colitis.^[1]^

The FDA adverse event reporting system (FAERS) is an essential database established to monitor the safety of drugs after they have been approved for public use. It serves as a crucial tool for post-marketing surveillance, ensuring that any potential risks or adverse effects associated with medications are identified and addressed. FAERS is a publicly accessible resource, making it a valuable asset for healthcare providers, researchers, and the general public who are interested in understanding the safety profiles of pharmaceutical products.^[3–6]^ The database includes a wide array of information, such as detailed reports on medication errors, patient demographics, adverse drug events, therapeutic outcomes, and other relevant clinical data. By collecting and organizing this information, the FDA can track patterns of adverse events and take appropriate actions to protect public health, including issuing safety warnings, revising drug labeling, or, in extreme cases, removing a product from the market.^[3–6]^ The current study aims to describe adverse events linked with third-generation cephalosporins using the FAERS Database.

## 2. Methodology

This was a retrospective, descriptive assessment of adverse events associated with third-generation cephalosporins submitted to the Food and Drug Administration (FDA). The study includes all reports that were submitted to the US FAERS before January 1, 2025. The reports submitted after December 31, 2024, were excluded from the study.

The data obtained for each medicine comprises the total number of reports made with FAERS, the gender and age of the patients who experienced adverse events, the reporters’ specializations, and the most common adverse occurrences. The data was evaluated descriptively and given as numbers and percentages in several tables. The tables contain data for third-generation cephalosporins (cefotaxime, ceftazidime, cefdinir, ceftriaxone, cefpodoxime, cefoperazone, and cefixime). An analysis was conducted using RevMan software to examine changes in adverse event reporting trends over the last 4 years, as well as the relationship between patient gender, age, reporter specialty, and serious adverse event rates, expressed as ORs with 95% CIs. AutoRegressive Integrated Moving Average (ARIMA) Model was used to find trends of reporting antibiotics adverse events over several years and to illustrate the actual and forecasted report counts for various antibiotics from 2015 to 2029.

No Institutional Review Board approval or informed consent was required for this research because the study relied solely on data that was publicly available and included only information about adverse events that had already been reported. This means the research involved secondary data analysis, where no new personal data or interventions were introduced, and the data was anonymized, protecting patient privacy. The adverse event reports used in the study were part of the FAERS, which is a publicly accessible database.

## 3. Results

**Figure 1.**
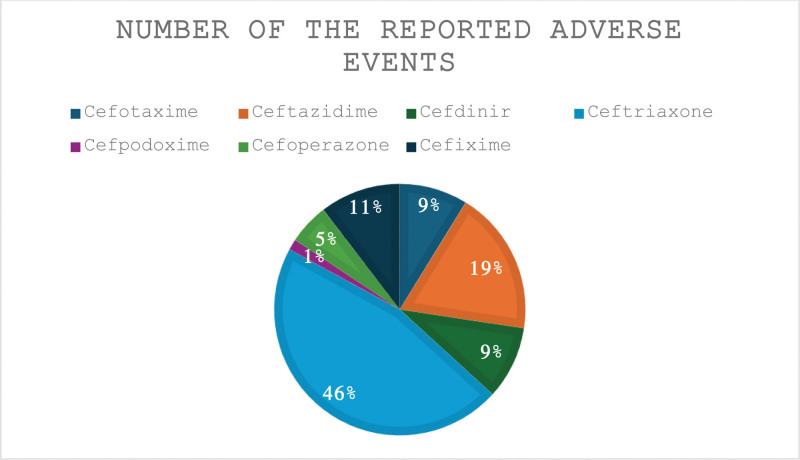
The total number of reported adverse events. This figure displays the total number of adverse events reported for third-generation cephalosporins. Data were obtained from the FAERS. FAERS = FDA adverse event reporting system.

Before January 1, 2025, 35,128 reports (3092 for cefotaxime, 6530 for ceftazidime, 3292 for cefdinir, 16,224 for ceftriaxone, 492 for cefpodoxime, 1839 for cefoperazone, and 3659 for cefixime) were submitted to the US FAERS (Fig. 1). Table 1 shows the reported adverse events of cefotaxime. Most of the reports were reported by healthcare professionals (88.42%). The ages of 48.80% of the patients who experienced adverse events were between 18 and 64 years, and 51.81% of them were females. The most reported adverse events of cefotaxime were pain (15.23%), eosinophilia (8.99%), ineffective drug (7.31%), pyrexia (6.11%), off-label use (4.04%), acute kidney injury (3.98%), toxic epidermal necrolysis (3.59%), and rash (3.36%).

**Table 1:** The reported adverse events of cefotaxime (n = 3092).

Variable	Category	Number	Percentage
Year of submission	2022–2024	566	18.31
	2019–2021	740	23.93
	2016–2018	332	10.74
	2013–2015	220	7.11
	Before 2013	1234	39.91
Age of the patients	<2 yr	295	11.21
	3–11	232	8.82
	12–17	155	5.89
	18–64	1284	48.80
	65–85	570	21.66
	>85	95	3.61
Gender of the patients	Male	1342	48.19
	Female	1443	51.81
Specialty of the reporters	Healthcare professional	2649	88.42
	Consumer	347	11.58
The reported adverse events	Pain	471	15.23
	Eosinophilia	278	8.99
	Ineffective drug	226	7.31
	Pyrexia	189	6.11
	Off-label use	125	4.04
	Acute kidney injury	123	3.98
	Toxic epidermal necrolysis	111	3.59
	Rash	104	3.36
	Thrombocytopenia	102	3.30
	Maculo-papular rash	97	3.14
	Aggravated condition	91	2.94
	Pruritus	88	2.85

Table 2 shows the reported adverse events of ceftazidime. Healthcare professionals reported the majority of the reports (87.09%). Of the patients who encountered adverse events, 57.17% were male and 47.97% were between 18 and 64. Ineffective medication (7.70%), pyrexia (5.70%), sepsis (5.65%), acute kidney injury (5.60%), shock (4.84%), and cardiac failure (4.62%) were the most frequently reported adverse effects of ceftazidime.

**Table 2:** The reported adverse events of ceftazidime (n = 6530).

Variable	Category	Number	Percentage
Year of submission	2022–2024	994	15.22
	2019–2021	1029	15.76
	2016–2018	643	9.85
	2013–2015	315	4.82
	Before 2013	3549	54.35
Age of the patients	<2 yr	211	3.64
	3–11	260	4.48
	12–17	231	3.98
	18–64	2783	47.97
	65–85	2098	36.17
	>85	218	3.76
Gender of the patients	Male	3468	57.17
	Female	2598	42.83
Specialty of the reporters	Healthcare professional	5507	87.09
	Consumer	816	12.91
The reported adverse events	Ineffective drug	503	7.70
	Pyrexia	372	5.70
	Sepsis	369	5.65
	Acute kidney injury	366	5.60
	Shock	316	4.84
	Cardiac failure	302	4.62
	Aggravated condition	296	4.53
	Pneumonia	250	3.83
	Off-label use	239	3.66
	Thrombocytopenia	228	3.49
	Death	224	3.43
	Toxic epidermal necrolysis	208	3.19

Table 3 shows the reported adverse events of cefdinir. Most of the reports were reported by healthcare professionals (58.16%). The ages of 36.61% of the patients who experienced adverse events were between 18 and 64 years, and 60.38% of them were females. The most reported adverse events of cefdinir were diarrhea (11.57%), drug hypersensitivity (11.57%), rash (8.60%), urticaria (6.38%), pyrexia (5.71%), and discolored feces (5.41%).

**Table 3:** The reported adverse events of cefdinir (n = 3292).

Variable	Category	Number	Percentage
Year of submission	2022–2024	411	12.48
	2019–2021	460	13.97
	2016–2018	441	13.40
	2013–2015	282	8.57
	Before 2013	1698	51.58
Age of the patients	<2 yr	462	20.16
	3–11	432	18.85
	12–17	120	5.23
	18–64	839	36.61
	65–85	391	17.06
	>85	48	2.09
Gender of the patients	Male	1093	39.62
	Female	1666	60.38
Specialty of the reporters	Healthcare professional	1771	58.16
	Consumer	1274	41.84
The reported adverse events	Diarrhea	381	11.57
	Drug hypersensitivity	381	11.57
	Rash	283	8.60
	Urticaria	210	6.38
	Pyrexia	188	5.71
	Discolored feces	178	5.41
	Vomiting	160	4.86
	Ineffective drug	148	4.50
	Nausea	137	4.16
	Pruritus	128	3.89
	Product storage error	102	3.10
	Dyspnea	94	2.86

Table 4 presents the adverse events associated with ceftriaxone. Healthcare professionals submitted the majority of these reports (96.17%). Among the patients who experienced adverse events, 47.72% were aged between 18 and 64 years, and 52.24% were males. The most frequently reported adverse events of ceftriaxone included off-label use (11.97%), ineffective drug (9.76%), eosinophilia (6.07%), acute kidney injury (4.86%), worsening of condition (4.82%), pyrexia (4.62%), and rash (4.14%).

**Table 4:** The reported adverse events of ceftriaxone (n = 16,224).

Variable	Category	Number	Percentage
Year of submission	2022–2024	4698	28.96
	2019–2021	5263	32.44
	2016–2018	2538	15.64
	2013–2015	1687	10.40
	Before 2013	2038	12.56
Age of the patients	<2 yr	532	3.83
	3–11	880	6.34
	12–17	384	2.77
	18–64	6621	47.72
	65–85	4470	32.21
	>85	989	7.13
Gender of the patients	Male	7571	52.24
	Female	6921	47.76
Specialty of the reporters	Healthcare professional	15,083	96.17
	Consumer	600	3.83
The reported adverse events	Off-label use	1942	11.97
	Ineffective drug	1583	9.76
	Eosinophilia	984	6.07
	Acute kidney injury	788	4.86
	Aggravated condition	782	4.82
	Pyrexia	750	4.62
	Rash	671	4.14
	Dyspnea	605	3.73
	Vomiting	526	3.24
	Pruritus	515	3.17
	Diarrhea	453	2.79
	Drug interaction	452	2.79

Table 5 presents the adverse events associated with cefpodoxime. Healthcare professionals submitted the majority of these reports (88.00%). Among the patients who experienced adverse events, 59.28% were aged between 18 and 64 years, and 58.06% were females. The most frequently reported adverse events of cefpodoxime included pyrexia (7.72%), pruritus (6.10%), diarrhea (5.08%), vomiting (4.88%), acute kidney injury (4.47%), and nausea (4.27%).

**Table 5:** The reported adverse events of cefpodoxime (n = 492).

Variable	Category	Number	Percentage
Year of submission	2022–2024	124	25.20
	2019–2021	109	22.15
	2016–2018	105	21.34
	2013–2015	100	20.33
	Before 2013	54	10.98
Age of the patients	<2 yr	48	13.30
	3–11	35	9.70
	12–17	24	6.64
	18–64	214	59.28
	65–85	20	5.54
	>85	20	5.54
Gender of the patients	Male	190	41.94
	Female	263	58.06
Specialty of the reporters	Healthcare professional	418	88.00
	Consumer	57	12.00
The reported adverse events	Pyrexia	38	7.72
	Pruritus	30	6.10
	Diarrhea	25	5.08
	Vomiting	24	4.88
	Acute kidney injury	22	4.47
	Nausea	21	4.27
	AGEP*	21	4.27
	Ineffective drug	21	4.27
	Aggravated condition	19	3.86
	Drug interaction	19	3.86
	Agranulocytosis	19	3.86
	Urticaria	19	3.86

* Acute generalized exanthematous pustulosis.

Table 6 outlines the adverse events linked to cefoperazone. Healthcare professionals submitted the majority of these reports (65.28%). Of the patients who experienced adverse events, 51.00% were between 18 and 64, and 56.50% were males. The most commonly reported adverse events of cefoperazone were a decreased prothrombin level (7.94%), hemorrhage (6.74%), pyrexia (6.63%), dermatitis (5.71%), thrombocytopenia (5.44%), and shock (5.22%).

**Table 6:** The reported adverse events of cefoperazone (n = 1839).

Variable	Category	Number	Percentage
Year of submission	2022–2024	30	1.63
	2019–2021	33	1.79
	2016–2018	23	1.25
	2013–2015	22	1.20
	Before 2013	1731	94.13
Age of the patients	<2 yr	73	5.04
	3–11	27	1.86
	12–17	28	1.93
	18–64	739	51.00
	65–85	503	34.71
	>85	79	5.45
Gender of the patients	Male	878	56.50
	Female	676	43.50
Specialty of the reporters	Healthcare professional	1160	65.28
	Consumer	617	34.72
The reported adverse events	Decreased prothrombin level	146	7.94
	Hemorrhage	124	6.74
	Pyrexia	122	6.63
	Dermatitis	105	5.71
	Thrombocytopenia	100	5.44
	Shock	96	5.22
	Abnormal hepatic function	91	4.95
	Clostridium colitis	84	4.57
	Sepsis	83	4.51
	Gastrointestinal hemorrhage	77	4.19
	Aggravated condition	73	3.97
	Death	73	3.97

Table 7 presents the adverse events associated with cefixime. Healthcare professionals submitted 39.84% of the reports. Among the patients who experienced adverse events, 39.68% were aged between 18 and 64, and 60.39% were females. The most frequently reported adverse events of cefixime included diarrhea (12.90%), Clostridium colitis (12.74%), ineffective drugs (11.23%), pyrexia (5.17%), dermatitis (4.95%), and urticaria (4.92%).

**Table 7:** The reported adverse events of cefixime (n = 3659).

Variable	Category	Number	Percentage
Year of submission	2022–2024	309	8.44
	2019–2021	452	12.35
	2016–2018	183	5.00
	2013–2015	149	4.07
	Before 2013	2566	70.13
Age of the patients	<2 yr	331	13.94
	3–11	438	18.45
	12–17	111	4.68
	18–64	942	39.68
	65–85	467	19.67
	>85	85	3.58
Gender of the patients	Male	1081	39.61
	Female	1648	60.39
Specialty of the reporters	Healthcare professional	1352	39.84
	Consumer	2042	60.16
The reported adverse events	Diarrhea	472	12.90
	Clostridium colitis	466	12.74
	Ineffective drug	411	11.23
	Pyrexia	189	5.17
	Dermatitis	181	4.95
	Urticaria	180	4.92
	Abdominal pain	152	4.15
	Vomiting	146	3.99
	Drug hypersensitivity	133	3.63
	Pruritus	103	2.81
	Arthralgia	83	2.27
	Nausea	82	2.24

**Figure 2.**
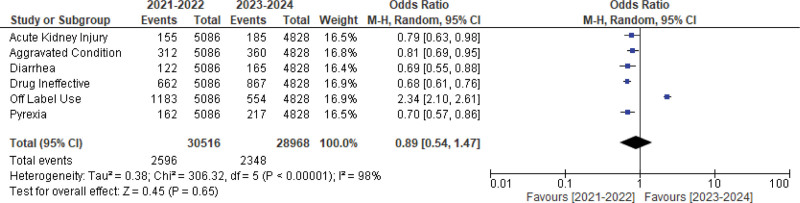
The difference in reporting the most reported adverse events in the last years. This figure illustrates the annual number of adverse event reports submitted from 2020–2024. A notable increase in overall reporting is observed during 2023–2024.

Table 8 highlights the changes in reporting trends for the most frequently reported adverse events in recent years. Over the last 2 years (2023–2024), there was an increase in reports of drug ineffective, pyrexia, diarrhea, acute kidney injury, and aggravated condition, while reports of off-label use decreased compared to the 2021 to 2022 period. Figure 2 illustrates that overall adverse event reporting rose in 2023 to 2024, though this increase was not statistically significant (OR: 0.89, CI: 0.54–1.47, *P*-value = .65).

**Table 8:** The difference in reporting the most reported adverse events in the last years.

Adverse events	2021–2022			2023–2024		
	Event	Total	Percent	Event	Total	Percent
Drug ineffective	662	5086	13.02%	867	4828	17.96
Off-label use	1183	5086	23.26%	554	4828	11.47
Pyrexia	162	5086	3.19%	217	4828	4.49
Diarrhea	122	5086	2.40%	165	4828	3.42
Acute kidney injury	155	5086	3.05%	185	4828	3.83
Aggravated condition	312	5086	6.13%	360	4828	7.46

The incidence of serious adverse events surpassed 90% for cefotaxime, ceftazidime, ceftriaxone, cefpodoxime, and cefoperazone. For cefixime, the rate was 81.01%, while cefdinir showed a lower rate of 47.02%. Cumulatively, 88.54% of all reported adverse events were serious (31,101 out of 35,128 cases) (Table 9).

**Table 9:** The number and percentage of the reported serious adverse events.

Variable	Number of serious adverse events	Total number of adverse events	Percentage of serious adverse events
Cefotaxime	3027	3092	97.90
Ceftazidime	6128	6530	93.84
Cefdinir	1548	3292	47.02
Ceftriaxone	15,215	16,224	93.78
Cefpodoxime	473	492	96.14
Cefoperazone	1746	1839	94.94
Cefixime	2964	3659	81.01
Total	31,101	35,128	88.54

**Figure 3.**
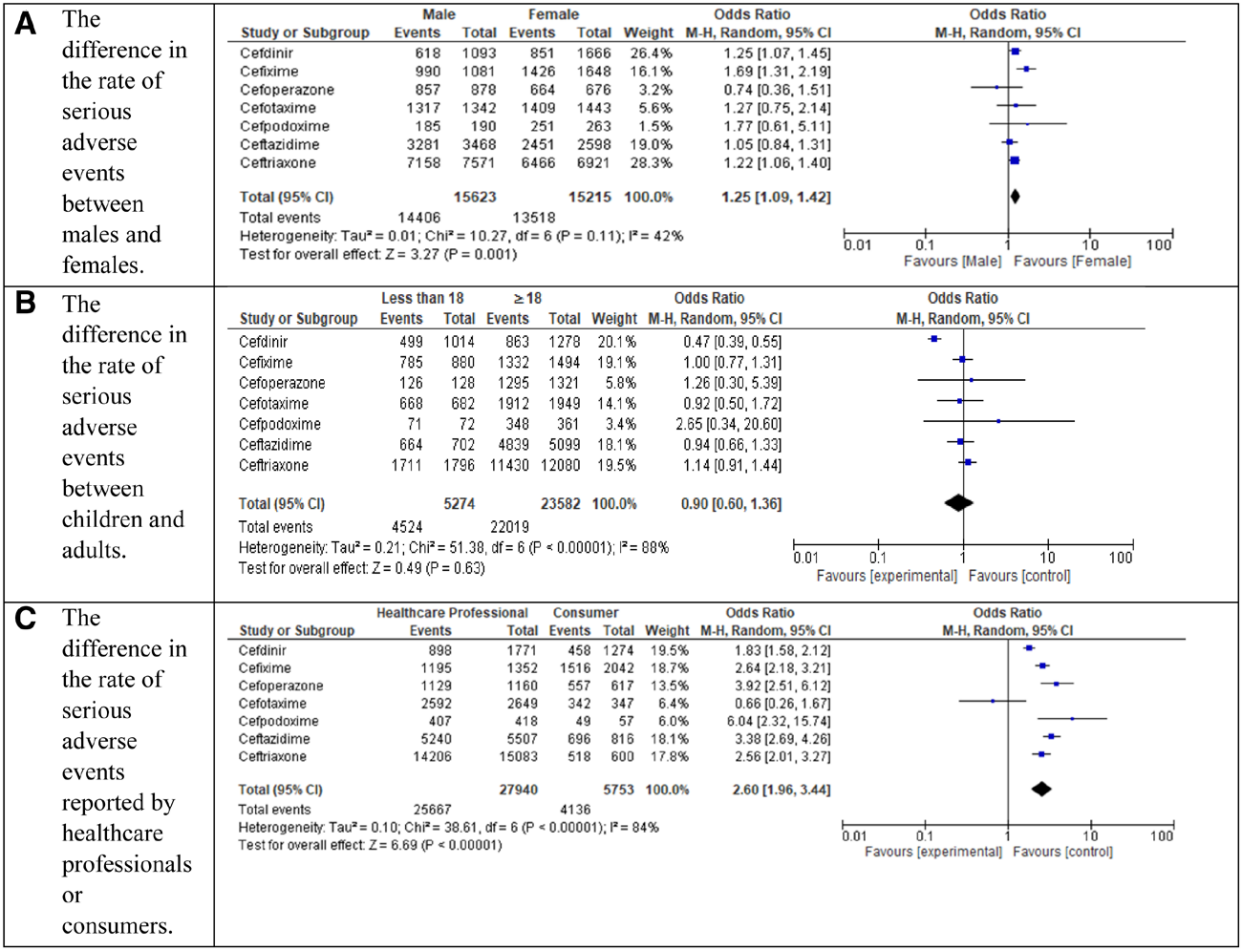
The relationship between patient gender, age, and reporter specialty with the incidence of serious adverse events. This figure explores the association between serious adverse event (SAE) incidence and key demographic and reporting variables, including patient gender, age group, and the specialty of the reporter.

Figure 3 demonstrates the relationship between patient gender, age, reporter specialty, and serious adverse event incidence, revealing that males had a significantly higher rate (92.21%, 14,406/15,623) compared to females (88.85%, 13,518/15,215) (OR: 1.25, 95% CI: 1.09–1.42, *P* = .001), while young patients showed a lower but non-significant rate (85.78%, 4524/5274) versus older patients (93.37%, 22,019/23,582) (OR: 0.90, 95% CI: 0.60–1.36, *P* = .63); additionally, reports from healthcare professionals had a markedly higher serious adverse event rate (91.86%, 25,667/27,940) than those from consumers (71.89%, 4136/5753) (OR: 2.60, 95% CI: 1.96–3.44, *P* < .00001).

To better understand longitudinal patterns in antibiotic-related ADR reporting, a time-series analysis was conducted using FAERS data. Given RevMan’s limitations for this type of analysis, Python was used to apply ARIMA modeling and forecast trends through 2029. This approach revealed non-linear changes in reporting rates, particularly for ceftriaxone, highlighting potential temporal associations with external health events. Incorporating such methods provides a more nuanced view of drug safety surveillance and allows healthcare systems to proactively respond to emerging ADR risks.

**Figure 4.**
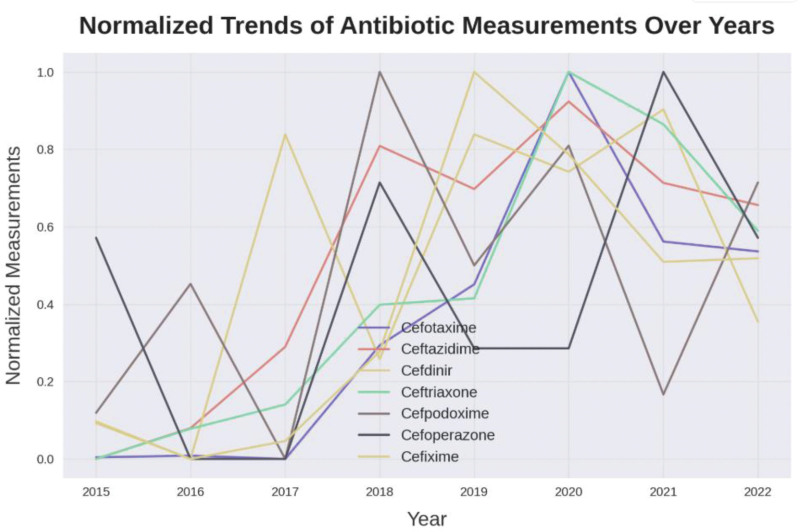
The normalized trends of antibiotic measurement over years. This figure illustrates the annual number of reported adverse events associated with selected antibiotics in the FAERS database. An increasing trend is observed over the years, indicating a rise in antibiotic-related safety concerns. Time-series analysis using the augmented Dickey–Fuller test revealed non-stationarity in the data, highlighting the need for advanced forecasting techniques such as ARIMA modeling to avoid spurious correlations. ARIMA = AutoRegressive Integrated Moving Average, FAERS = FDA adverse event reporting system.

Figure 4 shows the increased trends of reporting antibiotics adverse events over several years. Using ARIMA Model, the analysis showed that augmented Dickey–Fuller test results (statistic > critical values at all levels and high *P*-value > .10) indicate failure to reject the null hypothesis, meaning the time series is non-stationary and can lead to spurious correlations and poor forecasts.

**Figure 5.**
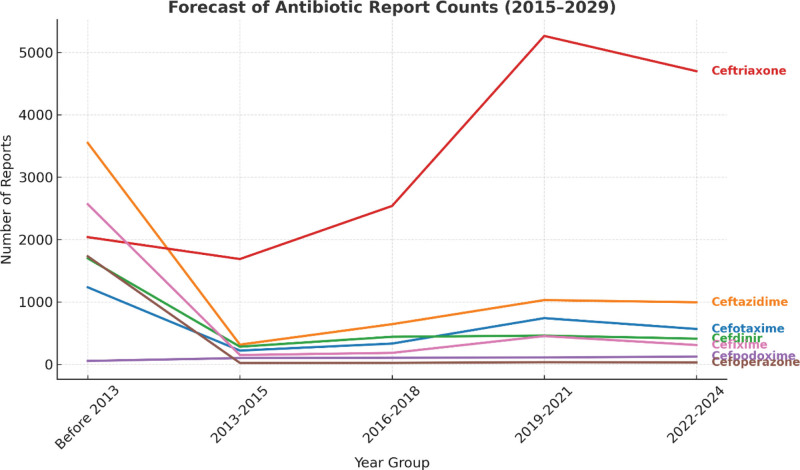
Forecast of antibiotic report counts (2015–2029). This figure presents both historical and forecasted adverse event report counts for selected antibiotics using the ARIMA model. Ceftriaxone shows the highest volume of reports, with a peak around 2020 followed by a decline and subsequent stabilization. Other antibiotics such as cefotaxime, ceftazidime, cefixime, cefpodoxime, cefoperazone, and cefdinir exhibit stable and lower reporting trends. The model forecasts minimal fluctuations in future reporting patterns, emphasizing the continued prominence of ceftriaxone in pharmacovigilance monitoring. ARIMA = AutoRegressive Integrated Moving Average

Figure 5 illustrates the actual and forecasted report counts for various antibiotics from 2015 to 2029. Ceftriaxone shows the highest number of reports, peaking above 2000 around 2020 before declining and stabilizing at approximately 1650 in the forecast period (2025–2029). cefotaxime and ceftazidime follow with moderate and relatively stable counts, while other antibiotics like cefixime, cefpodoxime, cefoperazone, and cefdinir maintain low and steady report levels. Overall, the forecast suggests minimal change in reporting trends for most antibiotics beyond 2024, with ceftriaxone continuing to dominate in usage or reporting frequency.

## 4. Discussion

Third-generation cephalosporins are a class of beta-lactam antibiotics widely used for their broad-spectrum activity, particularly against Gram-negative bacterial infections. While generally well-tolerated, these antibiotics are associated with several adverse effects, as evidenced by our analysis of the FAERS database. The most prominent concerns include antibiotic resistance, GI disturbances, and hypersensitivity reactions, each of which warrants careful consideration in clinical practice.

The most frequently reported issue with third-generation cephalosporins was drug ineffectiveness, primarily driven by the emergence of antibiotic resistance. The misuse or overuse of these antibiotics accelerates bacterial resistance, rendering them ineffective against certain infections. Resistance mechanisms are diverse and well-documented. For instance, Principe et al highlight that Haemophilus parainfluenzae can develop resistance through substitutions in PBP3 and PBP5.^[7]^ Additionally, extended-spectrum beta-lactamase (ESBL)-producing organisms, particularly E. coli, are becoming increasingly prevalent, as noted by Paterson and Bonomo and further supported by Park, who attributes this trend to the global spread of CTX-M-type ESBLs.^[8–11]^ Bush further emphasizes that ESBLs, AmpC β-lactamases, and OXA-type β-lactamases collectively contribute to the global threat of third-generation cephalosporin resistance, undermining modern antibacterial therapies.^[12]^ Given these findings, it is clear that combating resistance requires coordinated efforts at local, national, and international levels, including stricter antibiotic stewardship to minimize unnecessary prescriptions.

Beyond resistance, our study identified GI disturbances – such as diarrhea, nausea, and vomiting – as frequently reported side effects of third-generation cephalosporins. These findings align with previous literature. Arumugham et al note that cephalosporins can cause a range of adverse reactions, from mild GI symptoms to severe conditions like pseudomembranous colitis.^[2]^ Similarly, Mitropoulos et al and Choi et al report that nausea, vomiting, and diarrhea are among the most common adverse events, with skin reactions also being prevalent. Given the frequency of these effects, clinicians should monitor patients, especially those with preexisting GI conditions, and consider symptomatic management or alternative treatments when necessary.^[13,14]^

Another critical concern highlighted in our analysis is hypersensitivity, which, although rare, can have serious consequences. Moreno et al point out that while cephalosporin-related allergic reactions were historically considered uncommon, their increasing use has led to a rise in reported cases.^[15]^ Khan et al study further explains that these reactions stem from diverse immunopathologic mechanisms, with cephalosporins being a leading cause of severe cutaneous adverse events and perioperative anaphylaxis. Cross-reactivity with penicillin allergies is also a concern;^[16]^ Arumugham et al estimate that up to 10% of penicillin-allergic patients may react to cephalosporins due to structural similarities.^[2]^ Diagnostic challenges further complicate this issue – Touati et al found that nearly 25% of patients evaluated for cephalosporin hypersensitivity required drug provocation tests for confirmation, highlighting the need for improved diagnostic approaches.^[17]^ Given that Macy et al report a 1 to 3% prevalence of cephalosporin allergy in the general population, heightened awareness and careful patient screening are essential to mitigate risks.^[18]^

The findings reveal a strikingly high proportion of serious adverse events (88.54%, 31,101/35,128), underscoring potential safety concerns with the studied medications. Notably, male patients exhibited a significantly higher incidence of serious adverse events (92.21%) compared to females (88.85%; OR: 1.25, *P* = .001), suggesting possible gender-based differences in drug metabolism, reporting biases, or biological susceptibility. While younger patients had a numerically lower rate (85.78%) than older patients (93.37%), this difference lacked statistical significance (*P* = .63), possibly due to the confounding factors like comorbidities in older populations. Importantly, adverse events reported by healthcare professionals were substantially more likely to be classified as serious (91.86%) than those reported by consumers (71.89%; OR: 2.60, *P* < .00001), which may reflect differences in symptom recognition, reporting thresholds, or clinical severity.

There are several limitations to the study. Firstly, the study does not compare cephalosporins to other antibiotic classes (e.g., fluoroquinolones, beta-lactams), making it difficult to contextualize the findings. The study also does not provide a denominator (e.g., adverse events per 100,000 prescriptions), making the results difficult to interpret in terms of real-world risk. Moreover, since reporting is voluntary, there may be a bias toward more serious or unusual adverse events being reported. In contrast, mild or common adverse events may be underreported, skewing the results. Furthermore, in the FAERS, an antibiotic reported as “ineffective” typically means that the drug failed to treat the infection as expected. This often (but not always) implies antibiotic resistance, though FAERS alone cannot confirm resistance without additional clinical or lab data.

The study also presents individual adverse event rates per drug but does not analyze which cephalosporin has the highest overall risk profile. Additionally, the FAERS database does not provide direct evidence of causality between third-generation cephalosporins and adverse events. The data only reflect temporal associations, and causality cannot be definitively established without further clinical investigation, such as controlled trials or cohort studies. Moreover, a key limitation of this study is the reliance on ARIMA modeling to analyze adverse event trends, which primarily captures linear patterns based on historical data. This approach may not fully account for complex, multifactorial influences such as changes in drug availability, prescribing preferences, dosage variations, concomitant illnesses, or shifts in disease prevalence (e.g., increased antibiotic use during COVID-19). Additionally, pharmacogenomic factors and evolving clinical practices can impact adverse event rates but are not integrated into this model. Therefore, the ARIMA model’s predictive capacity may be limited to short-term trends and might not reflect sudden or non-linear changes in drug use or adverse event incidence. Future research incorporating hybrid models, including neural networks or other machine learning approaches, could better capture these complexities.

## 5. Conclusion

This descriptive analysis of FAERS data identified GI issues, hypersensitivity reactions, and inadequate therapeutic responses as the most frequently reported adverse events associated with third-generation cephalosporins. While these findings do not establish causality or comparative risk, they underscore the importance of vigilance when prescribing these antibiotics. Healthcare professionals should consider these potential adverse events in clinical decision-making, particularly for patients at higher risk for GI or hypersensitivity reactions. Further studies with controlled designs are needed to validate these observations and assess their clinical significance.

## 6. Clinical implications

Prescribers of third-gen cephalosporins should monitor GI effects (consider probiotics or shorter courses in high-risk patients) and watch for hypersensitivity, especially in penicillin-allergic individuals. Use alternatives/desensitization for high-risk cases. For treatment failures, confirm pathogens via cultures, check resistance, and adjust therapy. Patient education on side effects/adherence and antimicrobial stewardship improve outcomes. Report adverse events to FAERS for surveillance. The analysis of antibiotic reports counts from 2015 to 2029 reveals a persistently high volume of adverse drug reaction (ADR) reports for ceftriaxone, while other antibiotics show relatively stable and lower trends. This suggests a need for heightened pharmacovigilance, especially for high-report drugs, and presents an opportunity for healthcare professionals to play a more proactive role. Clinical pharmacists can help identify, document, and report ADRs more systematically, while nurses – being at the frontline of patient care – can recognize early signs of adverse reactions and ensure timely reporting. Enhancing FAERS reporting requires structured training for healthcare workers, integrating ADR documentation into electronic health records, and fostering a culture of safety and accountability. Public awareness campaigns and simplified reporting tools for patients can further strengthen post-marketing surveillance, ultimately improving drug safety and therapeutic outcomes.

## Acknowledgments

The authors extend their appreciation to Prince Sattam bin Abdulaziz University for funding this research work through the project number (PSAU/2024/03/31977).

## Author contributions

**Conceptualization:** Nehad J. Ahmed.

**Data curation:** Nehad J. Ahmed.

**Formal analysis:** Nehad J. Ahmed.

**Funding acquisition:** Nehad J. Ahmed.

**Investigation:** Nehad J. Ahmed.

**Methodology:** Nehad J. Ahmed.

**Project administration:** Nehad J. Ahmed.

**Resources:** Nehad J. Ahmed.

**Software:** Nehad J. Ahmed.

**Supervision:** Nehad J. Ahmed.

**Validation:** Nehad J. Ahmed.

**Visualization:** Nehad J. Ahmed.

**Writing – original draft:** Nehad J. Ahmed.

**Writing – review & editing:** Nehad J. Ahmed.
